# A new method for vessel segmentation based on a priori input from medical expertise in cine phase-contrast Magnetic Resonance Imaging

**DOI:** 10.1186/1532-429X-16-S1-P355

**Published:** 2014-01-16

**Authors:** Sebastian L Bidhult, Marcus Carlsson, Katarina Steding-Ehrenborg, Håkan Arheden, Einar Heiberg

**Affiliations:** 1 Department of Clinical Physiology, Lund University, Lund, Skane, Sweden; 2 Centre for Mathematical Sciences, Lund University, Lund, Skane, Sweden

## Background

Phase contrast magnetic resonance imaging (PC-MRI) is the current gold standard for blood flow quantification. Using this technique it is possible to quantify cardiac output, stroke volume, shunts and valve insufficiencies. The accuracy of flow quantification is highly dependent on the manual delineation of the vessels of interest. Therefore, the purpose of this study is to develop an automatic method for vessel segmentation in PC-MRI sequences.

## Methods

A total of 211 subjects from a previous study [1] were included (160 heart failure patients with ejection fraction below 40% and 51 healthy volunteers). For all subjects manual delineations of aorta ascendens were available in all timeframes. The subjects were randomly divided into a training (n = 40) and a test set (n = 171). The manual delineations in the training set were parameterized and principle component analysis (PCA) was performed to extract a priori information regarding vessel shape change and motion during the cardiac cycle. The proposed algorithm is an active contour model which tracks the edges of the aortic wall, initialized by a manual delineation in one timeframe. Every deformation is followed by a projection onto a subset of linearly independent components from the PCA result of the a priori dataset. This procedure is repeated 8 times. Validation was performed by comparing automated delineations with manual delineations in the test set consisting of the 171 remaining patients. All automatic segmentations were initiated with the manual delineation from the user in one timeframe. Stroke volume was used as performance measure. The inter observer variability for manual delineations from two observers was calculated in the previous study [1].

## Results

**Figure 1:**
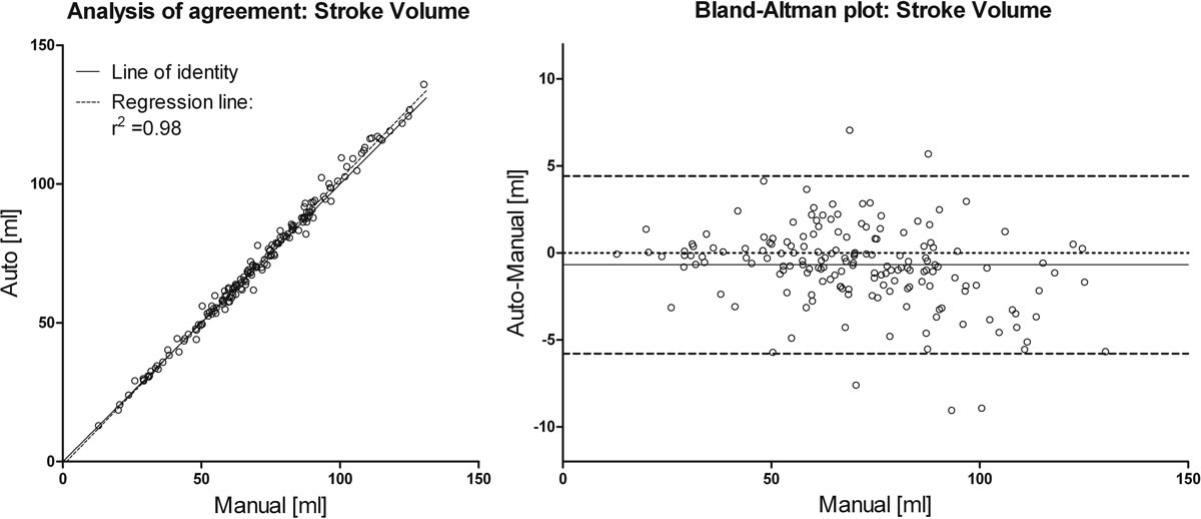
**Left: scatter plot of automatically measured stroke volume, and gold standard (manual delineation)**. Right: Bland-Altman plot of manual versus automated delineation. The dashed lines indicate ± 2SD and the solid line indicates bias between the two methods.

The stroke volume from the automated method agreed well with manual delineations (Figure 1), with a correlation coefficient of r2 = 0.98. Bias between automated and manual delineation was -0.69 ± 2.55 ml, or -0.7 ± 3.5% of the stroke volume. This should be compared to an inter observer variability of 3 ± 4% [1]. The low bias of the proposed method compared to the inter observer variability of the previous study [1] is likely attributed to the fact that the initialization of the automatic method was done by the observer to whom the comparison was made.

## Conclusions

In conclusion, the proposed method for vessel segmentation results in bias and variability comparable to manual delineations.

## Funding

Swedish Research Council, Swedish Heart-Lung Foundation, Medical Faculty at Lund University.
